# Effect of blanching time–temperature on potassium and vitamin retention/loss in kale and spinach

**DOI:** 10.1002/fsn3.4186

**Published:** 2024-04-26

**Authors:** Beatrice Muthoni Mugo, Juliana Kiio, Ann Munyaka

**Affiliations:** ^1^ Department of Foods Nutrition and Dietetics, School of Health Sciences Kenyatta University Nairobi Kenya

**Keywords:** blanching, end stage kidney disease, nutrient retention, potassium leaching

## Abstract

Hyperkalemia is common among patients with end stage kidney disease. Management involves diet modification. Hot water blanching is recommended to leach potassium in vegetables which results in losses of water‐soluble and heat labile vitamins. Evidence on the effect of blanching in reducing potassium level of locally consumed vegetables in Kenya is limited. This study sought to establish effect of hot water blanching time‐temperature on level of potassium, vitamin B1, B3 and C in kales (*Brassica oleracea var. acephala*) and spinach (*Spinach oleracea*) on potassium and vitamins B1, B3 and C retention/loss. The study adopted a full factorial experimental design. Vitamins were determined using high performance liquid chromatography. Potassium was quantified using atomic absorption spectrophotometry. To compare nutrient content between samples, independent t‐test and Analysis of Variance were used at 95% confidence level. Nutrient content of fresh kales and spinach were potassium (102 mg/100 g and 615 mg/100 g), vitamin B1 (124 μg/100 g and 51 μg/100 g), vitamin B3 (1165 μg/100 g and 812 μg/100 g) and vitamin C (102 mg/100 g and 116 mg/100 g) respectively. In kales, blanching for 20 min at 1000°C resulted to retention of 86.9%, 55.6%, 27.6% and 12.9% of vitamin B1, B3, C and potassium respectively. In spinach, blanching for 20 min at 1000°C resulted in retention of 79.9%, 88.6%, 12.2% and 40.6% retention of vitamin B1, B3, C and potassium respectively. Vitamin C and Potassium were the most sensitive to heat and leaching. Time had a greater effect than temperature in this study. This study recommends blanching of kale at 15.2 min at 800°C, spinach at 17.7 min at 840°C. Further research on optimal blanching time‐temperature for potassium and vitamin retention/loss is recommended.

## Graphical abstract

Blanching kale and spinach results in potassium loss in vegetables. Training ESKD patients on the recommended time to reduce potassium content in kale and spinach is required to allow a variety of vegetables in their diet.

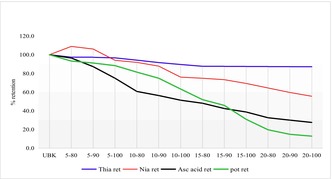

## 1 INTRODUCTION

Globally, there has been an alarming increase in the burden of chronic kidney disease (CKD) from 7.80 million in 1990 to 18.99 million in 2019. Incidence, death, and disability‐adjusted life years (DALYs) increased from 21.50 million to 41.54 million. (Jha & Modi, 2018). In Africa, the prevalence of hyperkalemia (<5.0 Mmol/L) is 15.8% in the general population and 32.8% in the high‐risk population. The prevalence of CKD of 17.7% is higher in sub‐Saharan Africa compared to that of 6.1% reported in North Africa (Kaze et al., 2018). In Kenya, the number of CKD patients has doubled since 2015, with a current hospital prevalence of 8% (Cherono, 2017). Hyperkalemia is common in CKD patients due to a decline in kidney function (Sarafidis et al., 2012). Hyperkalemia is defined as potassium serum levels above 5.0 mmol/L. Globally, there is a high prevalence of hyperkalemia among patients with CKD (Sanlier & Guler, 2018). The highest levels of hyperkalemia (5.1 mmol/L) in the general population were found in Europe, Australia, and New Zealand, compared with Japan (5.0 mmol/L) and North America (4.8 mmol/L) (Schellack et al., 2015). According to global reports, hyperkalemia among CKD patients is quite prevalent, at 40%–50% (Severini et al., 2016). However, there is a scarcity of literature on the prevalence of hyperkalemia among CKD patients in Kenya.

As part of Medical Nutrition Therapy (MNT) in End Stage Kidney Disease (ESKD), there is a need to control dietary potassium as a way of reducing hyperkalemia and its complications, which are frequently observed in these patients, which include bradycardia due to heart block or tachypnea due to respiratory muscle weakness, muscle weakness and flaccid paralysis, depressed or absent deep tendon reflexes (Fadupin et al., 2017). Patients with ESKD are advised to avoid foods high in potassium and encouraged to consume low‐potassium foods. To lower potassium from foods, patients are advised on their choice of foods and food preparation methods, especially blanching to leach potassium from foods. Blanching helps to reduce the potassium load in vegetables, but at the same time, it reduces the concentration of other nutrients (Cupisti et al., 2018). Patients with ESKD need to be guided by dieticians in planning a dietary self‐management plan to enable them to get the benefits of healthy, nutritious meals that contain adequate vitamins for their optimal overall health (Cupisti et al., 2018). Nutritional guidelines on blanching vegetables to attain low potassium and retain other water‐soluble nutrients are, however, lacking in Kenya.

Although potassium restriction in ESKD contradicts the recommendations for healthy diet practices for the control of non‐communicable diseases (NCDs), it is inevitable in the diets of ESKD patients in order to reduce the risk of hyperkalemia (Dunn et al., 2015). Leaching of potassium from food deprives ESKD patients of the benefit of other vitamins and minerals since they are also lost during blanching. The low‐potassium diets that are usually recommended for ESKD patients are also low in vitamin content, and the restriction lowers the dietary diversity (Fadupin et al., 2017). This has led to frequent cases of vitamin and mineral deficiencies among ESKD patients, which have not been adequately addressed (Stats, 2017). Patients with ESKD, especially those on hemodialysis, suffer low intakes of micronutrients, which are associated with diet restrictions, inadequate intake, and gastrointestinal changes that lower absorption. Prolonged insufficient intake of vitamins leads to micronutrient deficiencies among this population (Jankowska et al., 2017).

Green leafy vegetables (GLVs) are rich sources of vitamins and minerals, which play vital roles in the body. Green leafy vegetables are the best sources of carotenoids and polyphenols, which are important phytochemicals that scavenge free radicals (Macrae et al., 1993). Epidemiologically, increased intake of micronutrients is associated with a lower risk of non‐communicable diseases (NCDs), which is a major concern worldwide. However, some GLVs, such as kale and spinach, contain high levels of potassium (>300 mg/100 g), making them unsuitable for ESKD patients. Processing procedures, especially blanching of vegetables, have been employed to lower the potassium content in the diets of ESKD patients to make them suitable for this group of patients (Cupisti et al., 2018). Blanching vegetables lowers water‐soluble micronutrient content due to their tendency to leach out into water. The longer the time taken to blanch vegetables, the less the retention of water‐soluble micronutrients in the vegetables (Severi et al., 1997). Prolonged blanching time of vegetables reduces the content of the water‐soluble nutrients in the diet, with the losses having advantages and disadvantages for ESKD patients in that there is a reduction in hyperkalemia cases, but other important nutrients such as vitamin B complex and C are lost (Bamidele et al., 2017). Industrial blanching time and temperature are usually 70–100°C within 10–15 min as a pre‐treatment of food before preservation (Gonçalves et al., 2007).

According to a report from a study on the fresh vegetable market in Kenya, the most commonly consumed vegetables by the general population are kale (*Brassica oleracea var. acephala*) and spinach (*Spinacia oleracea*), which are consumed by 42% of the population (Research Solutions Africa‐RSA2015). Reasons cited for the high consumption of these vegetables are proximity, accessibility, price, and convenience. Reportedly, GLVs are cheap and readily available nutrient sources, especially in rural areas (Munyaka et al., 2010). Kale and spinach are rich sources of potassium, with the content being reportedly as 678 mg and 470 mg in 100 g dry matter, respectively (Nkafamiya et al., 2010; Steiber & Carrero, 2016). This study sought to investigate the effects of hot water blanching using different time–temperature combinations on potassium and vitamins B_1_, B_3_, and C retention/loss in kale and spinach.

## 2 METHODOLOGY

### 2.1 Research design

This study adopted a full‐factorial experimental research design to determine the effect of hot water blanching using different time/temperature treatment combinations on potassium and vitamins B_1_, B_3_, and C retention/loss in kale and spinach.

Fresh (unblanched) kale and spinach were used as controls in this study. The time and temperature combinations used as blanching treatments were blanching times of 5, 10, 15, and 20 min and blanching temperatures of 80, 90, and 100°C. The independent variables were blanching time and temperature. The dependent variables were the content and retention of vitamins B_1_, B_3_, and C, and potassium in fresh and blanched kale and spinach.

### 2.2 Sampling and preparation of samples

Makongeni market was randomly selected among Githurai, Wangige, Kangemi, and Kiambu markets in Nairobi and Kiambu, which are the most populous counties in Kenya. Fresh kale (*Brassica oleracea acephala*) and spinach (*Spinacia oleracea*) were sourced from randomly selected stalls at Makongeni Market in Thika. The samples were then transported in a cooler box to the Kenyatta University Food Chemistry Laboratory and stored at 4°C for a maximum of 2 days prior to blanching and nutrient analysis. The samples were sorted manually, washed, and rinsed using distilled water. This was followed by removing hard stalks and withered leaves, followed by the cutting of vegetables into small pieces. Samples of 50 g each were weighed and coded.

### 2.3 Determination of the moisture content of kale and spinach

Moisture content of fresh and blanched kale and spinach samples was conducted following the Association of Official Analytical Chemists (AOAC, 2005) International Method 970.30. The moisture content of kale and spinach was determined by drying 5 g of the vegetables at 105°C in a hot air oven (LDO‐08011, Japan) for 3 h until a constant weight was obtained, followed by cooling in a desiccator for 30 min before taking the weight of the samples. The calculation of moisture content was done as shown in the following equation:

$$\begin{array}{c} \%\text{moisture}=(\left(\text{weight of fresh sample}\pm \text{dish}\right)–\left(\text{weight of}\;\mathrm{dry}\;\text{sample}\pm \text{dish}\right)/\\ \;\text{Weight of fresh sample}+\text{dish})\times 100. \end{array}$$

### 2.4 Blanching treatment of kale and spinach

Samples of kale and spinach, 500 g each, were packed in perforated plastic zip lock bags and blanched for 5, 10, 15, 20 min at 80°C, 90°C, and 100°C using a thermostatic water bath (DK‐420, XMTD‐204, Japan). All the samples were cooled immediately after blanching by immersing in ice water for 5 min, then refrigerated at 4°C for a maximum of 2 days prior to analysis.

### 2.5 Determination of vitamins B_1_ and B_3_ in fresh and blanched kale and spinach

#### 2.5.1 Materials

This study was carried out on kale and spinach, which contain vitamins B_1_and B_3_. Vitamin B1 and B3 standards were from Sigma‐Aldrich (Germany). Other equipment and materials include HPLC (Shimadzu Corporation, Japan), electrical balance (PGB600, Japan), micropipettes, pipette stands, and distilled water.

#### 2.5.2 Preparation of samples

Vitamin B_1_ and B_3_ extraction was done by grinding vegetables using a mortar and pestle. Ground vegetables (0.100 g) were weighed, put in a volumetric flask (100 mL), and 80 mL of deionized water added. This was followed by ultrasonic extraction for 15 min, followed by topping to the mark with deionized water. The solution was then filtered using a 0.2 μm filter before being sonicated. The sample extracts were stored in dark glass containers to protect them from sunlight. These were then refrigerated at −20°C, awaiting analysis.

#### 2.5.3 Analytical conditions

A water symmetry C18 column (4.6 × 150 mm, 5 μm) was used for HPLC analysis; phosphate buffer (25 Nm) was prepared by dissolving 3.4 g of potassium dihydrogen phosphate and phosphoric acid in 1000 mL of distilled water to adjust PH to 7.0; florescence detector (SPD 20A) with extinction at a wave length of 266 nm was used for the detection of peaks. All analytical solutions were degassed by sonication prior to injection into the chromatographic system. All sample solutions were chromatographed so that interfering peaks were not present. Exactly 10 μL aliquots of the standard solution and sample extract were injected into the HPLC column and eluted at a flow rate of 0.8 mL per min.

#### 2.5.4 Estimation of B_1_ and B_3_

Concentration levels of 5, 10, 20, and 40 μg/mL were prepared from vitamins B_1_ and B_3_ of pure analytical grade (Merck KGaA, Germany) solutions by diluting using HPLC water. The 10 μL of vitamins B_1_ and B_3_ was injected into HPLC using autosampler and the analyses were monitored at 266 nm; the procedure was repeated three times. The average peak areas were plotted against the concentrations. The linearity of the method was then evaluated by using calibration curves to calculate coefficients of correlation, slopes, and intercept values. The content of the vitamins was calculated using the plotted peak areas of three samples of kale and spinach and the slope and intercept of the calibration curves of vitamins B_1_ and B_3_ standard in this equation: y=mx+c (Ekinci & Kadakal, 2005). The results were then multiplied by the dilution factor.

### 2.6 Determination of vitamin C in fresh and blanched kale and spinach

#### 2.6.1 Extraction of vitamin C

Kale and spinach (10 g each) were ground using a mortar and pestle before adding 50 mL of extraction solution. Metaphosphoric acid (MPA) of 3% concentration and acetic acid (8%) were used to prepare the extraction solution by adding 15 g of MPA to 40 mL of acetic acid and 200 mL of distilled water. Using Whatman filter paper no.1, the extract was filtered, put in a 100 mL volumetric flask, and topped up to the mark using extraction solution. Distilled water was used to top the solution to the mark in a 500 mL volumetric flask. Using the analytical balance model NBY323/64, 100 mg of vitamin C standard pure analytical grade (Merck KGaA, Germany) was weighed and put in a 100 mL beaker, and then dissolved with an extraction solution (45 mL). A 100‐mL volumetric flask was rinsed thrice using extraction solution, and the vitamin C standard solution was transferred into the flask, then topped up to the mark using extraction solution to make a concentration equal to 1000 ppm.

To prepare a standard curve, standards of different concentrations, that is, 10, 20, 40, 60, 80, and 100 ppm, were prepared by diluting the stock solution with extraction solution. The sample extract was centrifuged at 10,000*g*. This was followed by filtration (using a 0.45 μm membrane filter) of the supernatant and dilution using 10 mL of 0.8% MPA.

#### 2.6.2 Analytical conditions

The extraction of vitamin C was conducted using the method described by Macrae et al. (1993). Identification and quantification of vitamin C were performed using a reverse phase high‐pressure liquid chromatography machine (RP‐HPLC Shimadzu 20A, Kyoto, Japan), equipped with a RP‐HPLC column oven (LC‐20AD), a degasser (DGU‐20A5R), an LC pump (LU‐20AD), a UV–Visible diodidearray detector (SPD‐20A), and an autosampler (SIL‐20AHT). A reverse phase C_18_ column (Phenomenex C18, 250 × 4.6 mm, 5 μm particle size, Luna 5u) was used to carry out sample separation. The mobile phase was prepared by adding metaphosphoric acid (0.8%) at a flow rate of 1.2 mL/min set at 266 nm to monitor the effluent.

#### 2.6.3 Estimation of vitamin C

Standard curves were constructed by injecting standard solutions of 0.02, 0.04, 0.06, 0.08, and 0.1 mg/mL concentrations and observing the peak areas obtained. The standard and sample extract (20 μm) were injected into the RP‐HPLC column. The content of vitamin C was calculated using the plotted peak areas of three samples of kale and spinach.

### 2.7 Determination of potassium in fresh and blanched kale and spinach

#### 2.7.1 Sample preparation

Kale and spinach sample digestion and determination of potassium were conducted according to a previously described method (AOAC, 2005). Kale and spinach (2 g) were ground using a mortar and pestle, weighed using the analytical balance model NBY323/64, and put in a digesting tube (125 mL).

#### 2.7.2 Sample digestion

Ten milliliters of a solution of nitric acid and hydrochloric acid at a ratio of 3:1 were then added to the sample and homogenized using a hot plate stirrer (Wisestir MSH‐20A, Japan). The resulting mixture was then digested by heating on an electric hot plate (Digester, App No; 306765, Japan) at 90°C for 10 min. To avoid overheating, the flask was swirled, keeping the contents at the bottom. After removing the conical flask from the heater, the contents were left to cool, followed by the addition of 30% hydrogen peroxide drop by drop until the solution became clear. The digested sample was filtered, put in a volumetric flask (100 mL), and topped up to the mark using distilled water. The solution was filtered and put in a plastic container awaiting potassium analysis.

#### 2.7.3 Potassium quantification

Potassium was quantified by using a flame spectrophotometer (Sheewood 410 UK). The pure standard was sourced from Sigma Aldrich (Kenya). A 5 mL aliquot of the digested sample was pipetted into a 50 mL volumetric flask, followed by topping up to mark using deionized distilled water. Further dilution of 10 mL of diluted sample and blank was done at a ratio of 1:2. Standard solutions, samples, and blanks were randomly nebulized into the flame photometer. Solutions of different concentrations, that is, 1, 5, 10, 15, 20, and 25 mg/L, were injected into the flame photometer, and their resulting absorbance was used to construct standard curves. The concentration of potassium in the vegetable samples was determined by reading the concentration of the sample, which corresponds to its emission intensity from the calibration curve.

#### 2.7.4 Data analysis

All determinations were done in triplicate, and the mean values were calculated. SPSS statistical software for Windows version 22 was used to analyze the data, and results expressed as mean and standard deviation. To determine differences in the nutrient content between samples, an independent t‐test and Analysis of Variance (ANOVA) with a post‐hoc Least Significance of Difference (LSD) to separate the means were used. Regression analysis was used to determine the effect of temperature and time on nutrient content. The adequacy of each model was determined by evaluating the coefficient of determination (R^2^) and analyzing the residual plots to check for the normality assumption. True nutrient retentions were calculated according to Murphy et al. (1975) and expressed as a percentage using the following formula:

$$\begin{array}{c} \mathrm{TR}=(\text{nutrient content}\;\mathrm{per}\;\text{gram of food}\times \text{gram of food after cooking}/\\ \;\text{nutrient content}\;\mathrm{per}\;\text{gram of}\;\mathrm{raw}\;\text{food}\\ \;\times \text{gram of food before cooking})\times 100. \end{array}$$

## 3 RESULTS AND DISCUSSION

### 3.1 Potassium, vitamins B_1_ , B_3_, and C content in kale and spinach

The findings of the current study (Table 1) show that kale and spinach are rich in potassium and vitamins B_1_, B_3_, and C (Šamec et al., 2019; Sanlier & Guler, 2018). Vitamin B_1_ plays a role in energy metabolism, maintaining functions of the nervous system, strengthening the immune system, and maintaining a normal appetite (Schellack et al., 2015). Kale had a higher content of vitamins B_1_ and B_3_ compared to spinach, while spinach was richer in vitamin C and potassium compared to kale. Except for vitamin C, these findings are consistent with values reported in the literature (Agarwal et al., 2017; Haytowitz et al., 2019). The potassium content of spinach in the current study was 615.80 mg/100 g which was closer to values reported by other authors 558.00 mg/100 g (Haytowitz et al., 2019) and 502.00 mg/100 g. However, some studies reported a much lower value (5.40 mg/100 g) for potassium in spinach (Fadupin et al., 2017; Haytowitz et al., 2019). Potassium plays a vital role in the contraction of heart muscle, controls blood pressure, ensures optimal water balance, and balances the PH of the blood. The content of vitamin B_1_ (0.05 mg/100 g) observed in spinach was lower than 0.08 mg/100 g posted in the USDA, Food Composition Data Base and 0.08 mg/100 g in the West African Food Composition Table (WAFCT), but lower than 0.03 mg/100 g reported in the Kenya Food Composition Table (KFCT). The value of vitamin B_3_ content of spinach observed in the current study (0.51 mg/100 g) compares favorably with that of USDA (0.72 mg/100 g) but is higher than that of KFCTs (0.40 mg/100 g). The value of 116.00 ± 0.0 mg/100 g of vitamin C determined in spinach in the current study was way above those reported in KFCTs (37.00 mg/100 g) and the USDA (28.00 mg/100 g). Vitamin B_1_ levels of 0.11 mg/100 g in kale have been reported in the literature, which fall slightly below the values observed in the current study (Haytowitz et al., 2019; Pervin et al., 2017). The vitamin B_3_ content of 1165.00 mg/100 g observed in the current study compares well with values posted in the American nutrients database (1.18 mg/100 g), but is slightly higher than the values of 1.00 mg/100 g reported by both Sanlier and Guler (2018) and the KFCTs. The current study has established a vitamin C content of 102.21. Vitamin C determines many aspects of human health, regulating many metabolic processes. Vitamin C plays a role in iron absorption, cell growth and regeneration, synthesis collagen, and the formation of neurotransmitters. The values of 116.4 mg/100 g of vitamin found in the current study were way above those reported in the KFCTs (37.00 mg/100 g) and the USDA (28.10 mg/100 g). The current study established a vitamin C content of 102.21 in kale, which falls below 123.40 mg/100 g and 120.00 mg/100 g (Agarwal et al., 2017; Šamec et al., 2019), but is higher than the values found in the USDA nutrient database. Scientific evidence shows that the content of micronutrients in vegetables is influenced by their maturity at harvest, method of storage, and agronomic conditions of cultivation (Acikgoz & Deveci, 2011; Maraj et al., 2018; Munyaka et al., 2009; Pervin et al., 2017).

**TABLE 1:** The contents of potassium and vitamins B_1_, B_3_, and C in fresh kale and spinach.

Nutrient	Mean content ± SD	
	Kale	Spinach
Vitamin B_1_ (μg/100 g)	124.00 ± 0.1	51.10 ± 0.2
Vitamin B_3_ (μg/100 g)	1165.40 ± 0.1	811.70 ± 0.1
Vitamin C (mg/100 g)	102.33 ± 0.01	116.39 ± 0.01
Potassium (mg/100 g)	102.21 ± 0.12	615.80 ± 0.10

### 3.2 Effect of blanching conditions on potassium, vitamins B_1_ , B_3_, and C retention in kale and spinach

Statistical analysis using ANOVA indicated significant differences in the contents of micronutrients (potassium, vitamins B_1_, B_3_, and C) between fresh and blanched samples of both kale and spinach (*p* < .05) (Tables 2 and 3). Furthermore, the magnitude of change was dependent on the specific time–temperature conditions that were employed during the blanching process. Increasing either or both the time and temperature parameters resulted in a corresponding decrease in the micronutrient content of the vegetables. Similar findings about the effect of increasing blanching time and temperature on the micronutrient contents of vegetables have been reported (Kaur et al., 2018; Kawashima & Valente Soares, 2005; Munyaka et al., 2009; Natesh et al., 2017).

**TABLE 2:** Effects of different time–temperature blanching treatments on potassium, vitamins B_1_, B_3_, and C content of kale.

Sample	Vitamin B_1_ μg/100 g	Vitamin B_3_ μg/100 g	Vitamin C mg/100 g	Potassium mg/100 g
UBK	124.00 ± 0.01^a^	1165.04 ± 0.01^a^	102.33 ± 0.01^a^	102.21 ± 0.12^a^
BK 5‐80	135.10 ± 0.01^b^	1135.30 ± 0.01^b^	98.76 ± 0.01^b^	95.25 ± 0.00^b^
BK 5‐90	131.60 ± 0.01^c^	1133.30 ± 0.01^c^	89.76 ± 0.01^c^	93.40 ± 0.51^c^
BK 5‐100	116.80 ± 0.01^d^	1126.80 ± 0.01^d^	76.64 ± 0.01^d^	90.27 ± 0.00^d^
BK 10‐80	114.40 ± 0.03^e^	1098.70 ± 0.01^e^	62.15 ± 0.01^e^	83.13 ± 0.00^e^
BK 10‐90	108.90 ± 0.03^f^	1068.30 ± 0.01^f^	57.66 ± 0.01^f^	76.47 ± 0.00^f^
BK 10‐100	94.40 ± 0.01^g^	1043.30 ± 0.06^g^	52.56 ± 0.01^g^	64.70 ± 0.01^g^
BK 15‐80	92.90 ± 0.00^h^	1021.80 ± 1.00^h^	49.24 ± 0.01^h^	53.10 ± 0.00^h^
BK‐15‐90	91.50 ± 0.01^i^	1021.00 ± 1.00^i^	43.45 ± 0.01^i^	46.80 ± 0.01^i^
BK 15‐100	86.10 ± 0.01^j^	1018.50 ± 0.01^j^	39.64 ± 0.01^j^	31.56 ± 0.00^j^
BK 20‐80	80.00 ± 0.01^k^	1017.30 ± 0.01^k^	33.34 ± 0.01^k^	20.12 ± 0.00^k^
BK 20‐90	74.20 ± 0.02^L^	1016.40 ± 0.01^L^	30.72 ± 0.00^L^	15.33 ± 0.29^L^
BK 20‐100	70.80 ± 3.00^m^	1012.90 ± 0.01^m^	28.26 ± 0.00^m^	13.24 ± 0.00^m^

*Note*: Values are means ± SD of the mean. Superscripts with different letters in the same column indicate significantly different values at .05.

Abbreviations: BK, blanched kale; UBK, unblanched kale.

**TABLE 3:** Effects of different time–temperature treatments on vitamin potassium, B_1_, B_3_, and C content of spinach.

Sample	Vitamin B_1_ μg/100 g	Vitamin B_3_ μg/100 g	Vitamin C mg/100 g	Potassium mg/100 g
UBS	51.10 ± 0.02^a^	811.70 ± 0.01^a^	116.40 ± 0.01^a^	615.80 ± 0.01^a^
BS 5‐80	49.20 ± 0.01^b^	750.80 ± 0.01^b^	86.70 ± 0.01^b^	589.30 ± 0.00^b^
BS 5‐90	48.90 ± 0.01^c^	739.30 ± 0.01^c^	82.70 ± 0.01^c^	447.00 ± 0.00^c^
BS 5‐100	48.40 ± 0.01^d^	738.90 ± 0.01^bc^	76.80 ± 0.01^d^	403.40 ± 0.00^d^
BS 10‐80	46.70 ± 0.01^e^	744.10 ± 11.05^bc^	69.20 ± 0.01^e^	353.80 ± 0.00^e^
BS 10‐90	46.20 ± 0.01^f^	736.40 ± 0.01^cd^	61.60 ± 0.01^f^	326.40 ± 0.03^f^
BS 10‐100	45.30 ± 0.01^g^	731.50 ± 0.01^d^	52.70 ± 0.01^g^	307.80 ± 0.00^g^
BS 15‐80	44.70 ± 0.01^h^	729.90 ± 0.01^d^	43.30 ± 0.01^h^	302.60 ± 0.00^h^
BS 15‐90	44.20 ± 0.00^i^	728.70 ± 0.01^d^	37.60 ± 0.04^i^	299.40 ± 0.00^i^
BS 15‐100	43.90 ± 0.00^j^	727.10 ± 0.01^d^	25.70 ± 0.01^j^	298.30 ± 0.05^j^
BS 20‐80	42.70 ± 0.01^k^	726.80 ± 0.01^d^	18.60 ± 0.02^k^	297.80 ± 0.00^k^
BS 20‐90	41.60 ± 0.01^L^	724.20 ± 0.02^d^	15.30 ± 0.02^L^	282.40 ± 0.00^L^
BS 20‐100	40.80 ± 0.01^m^	718.80 ± 0.01^j^	14.20 ± 0.03^m^	250.10 ± 0.00^m^

*Note*: Values are means ± SD of the mean. Superscripts with different letters in the same column indicate significantly different values at .05.

Abbreviations: BS, blanched spinach; UBS, unblanched spinach.

**FIGURE 1:**
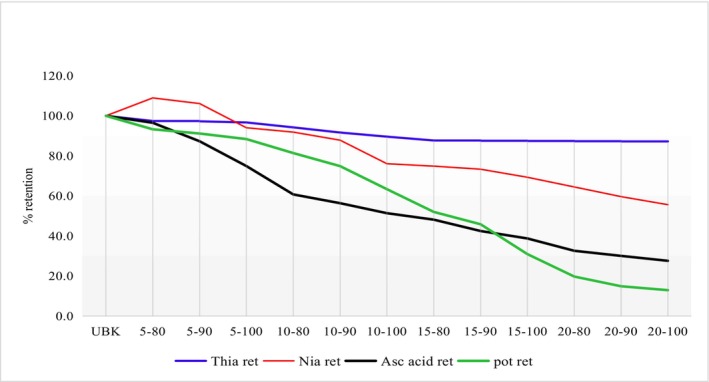
Potassium, vitamins B_1_, B_3_, and C in kale samples under different time–temperature blanching conditions.

Potassium was most susceptible to blanching treatments in kale, while vitamin B_1_ was the most stable nutrient (Figure 1). The extraction of vitamins and minerals into the cooking water is the major cause of loss rather than their destruction by heat, and potassium is probably the most sensitive mineral to this type of loss (Schiffmann et al., 2017). Therapeutic modification has been suggested as an option to control hyperkalemia (Dunn et al., 2015). Water‐soluble and heat‐labile nutrients leach out in water and are destroyed by heat during hot water blanching (HWB) treatment (Fadupin et al., 2017; Gupta et al., 2008; Ogbede et al., 2015). This could explain high losses of potassium in the vegetables. To reduce the complications of hyperkalemia in ESKD patients, blanching vegetables is recommended to reduce potassium (Cupisti et al., 2018). Vitamin C loss was also relatively high compared to the B vitamins. This could be attributed to both the effect of temperature and leaching during blanching.

**FIGURE 2:**
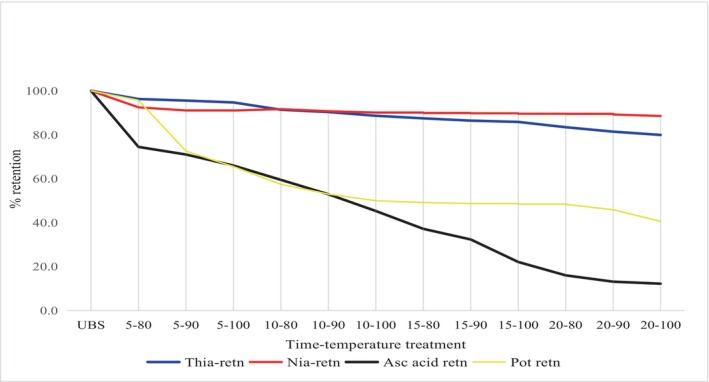
Potassium, vitamins B_1_, B_3_, and C in spinach samples under different time‐ temperature blanching conditions.

In this study, the highest loss of nutrients was observed for vitamin C, followed by potassium, vitamins B_1_ and B_3_ in spinach (Figure 2). Vitamin C was the least stable of the four nutrients, while vitamin B_3_ was the most stable during blanching. Vitamin C is reported to be the least stable of all vitamins and is easily destroyed during heat processing (Bergström, 1994). The current study determined a vitamin C retention of 4.3% at a temperature of 100°C for 20 min of blanching, compared to the aforementioned study, which reported vitamin C retention of 23.7%, 17.4%, and 16.7% during blanching at 80°C for 1, 2, and 4 min, respectively (Bergström, 1994). The treatment duration should be optimized to preserve nutrients and minimize nutrient losses in vegetables, particularly B vitamin and vitamin C, which play a crucial role in human health but are sensitive to processing treatments (Traoré et al., 2017). Functional vitamin B_1_ deficiency in ESKD patients may cause impaired energy metabolism, cardiovascular disease, anemia, oxidative stress, and increased mortality. Several dehydrogenases use vitamin B_3_ as a coenzyme for hydrogen transfer (Schellack et al., 2015).

Vitamin C sensitive to heat and leaching compared to B vitamins in both kale and spinach, with vitamin C retention of 45.1% (kale) and 4.3% (spinach) compared to vitamin B_1_ 83.9% (kale) and 70.7% (spinach) and B_3_ 84% (kale) and 89.7% (spinach) observed in the current study. There was a remarkable loss of potassium during blanching, where the percentage retention was 12.9% and 40.6% in kale and spinach, respectively. The trends in reduction of potassium and vitamin C are almost similar, in that the longer the blanching time and temperature, the higher the nutrient losses. The different leaching levels of potassium loss and vitamin C in kale and spinach could be attributed to the food matrix effects.

The current findings further demonstrate that the retention of potassium and vitamin C in kale and spinach is significantly lost (*p* < .05) by the combined effect of heat and leaching. An increase in blanching time resulted in increased nutrient loss in both kale and spinach (Tables 2 and 3 and Figures 1 and 2).

It is also notable that the trends in the loss of vitamin C were similar to those of potassium with an increase in time and temperature. This demonstrates that vitamin C is generally the most sensitive vitamin to heat and leaching compared to vitamins B_1_ and B_3_, whereas potassium is also sensitive but more stable than vitamin C.

Regression analysis for each of the test nutrients shows that time (X_1_) had a greater contribution to nutrient variation during blanching than temperature (X_2_) in both kale and spinach. Furthermore, for each minute increase in blanching time, there was loss of potassium (Y_1_), vitamin B_1_ (Y_2_), vitamin B_3_ (Y_3_) and vitamin C (Y_4_) content in kale by 5.00 mg/100 g, 7.76 μg/100 g, 299.00 μg/100 g, and 3.60 mg/100 g, respectively, while for each unit (°C) rise in temperature (X_2_), there was loss in potassium, vitamins B_1_, B_3_, and C content in kale by 110.00 mg/100 g, 0.10 μg/100 g, 0.098 μg/100 g, and 82.00 mg/100 g, respectively. On the other hand, for each minute increase in blanching time, there was a loss of potassium, vitamins B_1_, B_3_, and C content of spinach by 12.17 mg/100 g, 0.45 μg/100 g, 1.40 μg/100 g, and 4.35 mg/100 g, respectively. Similarly, for each unit (°C) rise in temperature, there was a loss in potassium, vitamin B_1_, B_3_, and vitamin C content in spinach by 1.52 mg/100 g, 0.00 μg/100 g, 0.65 μg/100 g, and 191.90 μg/100 g, respectively.

These findings are in agreement that there is a significant loss of potassium content with a successive 10 min increase in blanching time at 100°C (Fadupin et al., 2017). In a study on the effect of blanching time on selected mineral element extraction from the spinach substitute (*Tetragonia expansa*) commonly consumed in Brazil, potassium leaching losses of 19.00 mg/100 g, 35.00 mg/100 g, and 53.00 mg/100 g were observed with successive increases in blanching time of 1, 5, and 15 min, respectively, which corroborates the results obtained by the current study (Kawashima & Valente Soares, 2005).

The longer the blanching time, the greater the loss of micronutrients (Fadupin et al., 2017). At 5 min of blanching kale, there was 74.9% and 88.4% retention of vitamin C and potassium, respectively, compared to spinach, where the retention was 66% and 65.5%, respectively. After blanching for 10 min, retention of vitamin C and potassium was 51.4%, 63.3%, and 45.3%, 50% in kale and spinach respectively. After a further increase in time (15 min), vitamin C and potassium retention were 38.7%, 30.9% and 22.1%, 48.1%, respectively, in both vegetables. Maximum loss of both vitamin C and potassium was observed at 20 min, where the retention of both nutrients was 27.6%, 12.9%, and 12.2%, 40.6% in kale and spinach respectively (Tables 2 and 3). The difference between different nutrient retentions in both vegetables may be attributed to where they are placed in the matrix. The aim of this study was to explore potassium loss/vitamin retention in kale and spinach subjected to different blanching times and temperatures. As observed, it is inevitable to achieve maximum potassium loss without nutrient loss since some nutrients are water‐soluble and are lost in blanching water and, at the same time, destroyed by heat.

## 4 CONCLUSIONS

The current study established that blanching treatments resulted in a significant loss/retention of potassium, vitamins B_1_, B_3_, and C in both kale and spinach. Vitamin loss due to blanching was higher for vitamin C compared to both vitamin B_1_ and B_3_. The trends in potassium and vitamin C loss are almost similar, in that the longer the blanching time and temperature, the higher the nutrient losses. The effect of time was more pronounced than the temperature effect. Blanching kale for 15.2 min at a temperature of 80°C resulted in 33.7%, 83.9%, 70.7%, and 45.1% retention of potassium, vitamins B_1_, B_3_, and C, respectively, whereas blanching spinach for 17.7 min at a temperature of 84°C resulted in 47%, 84%, 89.7%, and 4.3% of potassium, vitamins B_1_, B_3_, and C, respectively.

## AUTHOR CONTRIBUTIONS

**Beatrice Muthoni Mugo:** Conceptualization (lead); data curation (lead); formal analysis (lead); investigation (lead); methodology (lead); resources (lead); software (lead); supervision (equal); validation (lead); visualization (lead); writing – original draft (lead); writing – review and editing (lead). **Juliana Kiio:** Conceptualization (equal); data curation (equal); formal analysis (equal); funding acquisition (supporting); investigation (supporting); methodology (supporting); project administration (supporting); resources (supporting); software (supporting); supervision (supporting); validation (supporting); visualization (supporting); writing – original draft (supporting); writing – review and editing (supporting). **Ann Munyaka:** Conceptualization (equal); data curation (equal); formal analysis (equal); funding acquisition (supporting); investigation (supporting); methodology (supporting); project administration (supporting); resources (supporting); software (supporting); supervision (supporting); validation (supporting); visualization (supporting); writing – original draft (supporting); writing – review and editing (supporting).

## CONFLICT OF INTEREST STATEMENT

The authors have no conflict of interest to declare for this study.

## Data Availability

All data are available in the main text.
